# The consumption of alcohol-based handrub during the 2009 influenza pandemic

**DOI:** 10.1186/1753-6561-5-S6-P105

**Published:** 2011-06-29

**Authors:** A Iten, N Vernaz, M Descombes, C Posfay Barbe, B Martinez De Tejeda Weber, L Kaiser, D Pittet

**Affiliations:** 1 Infection Control Program, University of Genva Hospitals, Geneva, Switzerland; 2 Pharmacy, University of Genva Hospitals, Geneva, Switzerland; 3 Department of Pediatrics, University of Genva Hospitals, Geneva, Switzerland; 4 Department of Obstetrics and Gynecology, University of Genva Hospitals, Geneva, Switzerland; 5 Central Laboratory of Virology, University of Genva Hospitals, Geneva, Switzerland

## Introduction / objectives

When the influenza A (H1N1) 2009 pandemic began, the Federal Office of Public Health released and regularly updated recommendations for the Swiss population and healthcare workers. At the University of Geneva Hospitals, the recommendations for patients, staff and visitors were adjusted according to the local epidemiology, hand hygiene and mask practices were reinforced, and screening of the cases and the prescription of the antiviral treatment were performed as indicated.

## Methods

Using interventional time-series analyses, we performed a transfer model with aggregated data on alcohol-based handrub (ABHR) in litres at HUG and the number of confirmed cases of influenza A (H1N1) in Switzerland as an indicator to evaluate the adherence to the recommendations from April 2009 to January 2010 on a weekly basis.

## Results

A statistically significant temporal relationship was found between the ABHR consumption and the number of H1N1 cases. Each additional H1N1 case was preceded by an increase of 0.51 ABHR liters at HUG (P<0.0001) on week earlier. The R2 coefficient was 96% expressing how close the observed values are to the fitted values generated by the estimated model.

## Conclusion

This study shows that modelling is a useful tool, complementing traditional epidemiologic approaches, can inform policy makers about the adherence to recommendations and could be used as an indicator in the follow-up of future influenza epidemics.

## Disclosure of interest

None declared.

